# Supplementary material for: Hybrid Models and Biological Model Reduction with PyDSTool
Source: PLoS Comput Biol. 2012 Aug 9;8(8):e1002628. doi: 10.1371/journal.pcbi.1002628 (PMC3415397; doi:10.1371/journal.pcbi.1002628)
Supplement: Text S4 — Complete source code for the PyDSTool package (version 0.88.120504). Includes API documentation and help files linking to web pages. This file is identical to the current public release on Sourceforge.net. (ZIP) [file pcbi.1002628.s004.zip › PyDSTool/html/PyDSTool.Model.HybridModel-class.html]

xml version="1.0" encoding="ascii"?


PyDSTool.Model.HybridModel


| Home | Trees | Indices | Help | PyDSTool |
| --- | --- | --- | --- | --- |

Package PyDSTool ::
Module Model ::
Class HybridModel

[hide private]

[frames] | no frames]

# Class HybridModel

source code

```
object --+    
         |    
     Model --+
             |
            HybridModel
```

---

obsvars specifies the observable variables for this model, which must
be present in all trajectory segments (regardless of which other
variables are specified in those segments). intvars specifies
non-observable (internal) variables for the model, which may or may not
be present in trajectory segments, depending on reductions, etc., that
are present in the sub-model (e.g. generator) that determines the
trajectory segment.


|  |  |
| --- | --- |
| Instance Methods | [hide private] |

|  |  |
| --- | --- |
| \_\_init\_\_(self, \*a, \*\*kw)  x.\_\_init\_\_(...) initializes x; see x.\_\_class\_\_.\_\_doc\_\_ for signature | source code |

|  |  |
| --- | --- |
| \_prepareICs(self, xdict, traj, ds, t0, ti\_1) | source code |

|  |  |
| --- | --- |
| \_applyStateMap(self, epochStateMaps, model\_interface, next\_model\_interface, traj, xdict, t0) | source code |

|  |  |
| --- | --- |
| cleanupMemory(self)  Clean up memory usage from past runs of a solver that is interfaced through a dynamic link library. | source code |

|  |  |
| --- | --- |
| \_findTrajInitiator(self, end\_reasons, partition\_num, t0, xdict, mi=None, swRules=None) | source code |

|  |  |
| --- | --- |
| \_addTraj(self, trajname, trajseq, epochEvents, modelNames, force\_overwrite=False)  Add a computed trajectory to database. | source code |

|  |  |
| --- | --- |
| haveJacobian(self)  Returns True iff all objects in modelInfo have defined Jacobians. | source code |

|  |  |
| --- | --- |
| haveJacobian\_pars(self)  Returns True iff all objects in modelInfo have defined Jacobians. | source code |

|  |  |
| --- | --- |
| Rhs(self, dsName, t, xdict, pdict=None, asarray=False)  Direct access to a sub-model generator's Rhs function. | source code |

|  |  |
| --- | --- |
| Jacobian(self, dsName, t, xdict, pdict=None, asarray=False)  Direct access to a sub-model generator's Jacobian function (if defined). | source code |

|  |  |
| --- | --- |
| JacobianP(self, dsName, t, xdict, pdict=None, asarray=False)  Direct access to a generator's JacobianP function (if defined). | source code |

|  |  |
| --- | --- |
| MassMatrix(self, dsName, t, xdict, pdict=None, asarray=False)  Direct access to a generator's MassMatrix function (if defined). | source code |

|  |  |
| --- | --- |
| AuxVars(self, dsName, t, xdict, pdict=None, asarray=False)  Direct access to a generator's auxiliary variables definition (if defined). | source code |

|  |  |
| --- | --- |
| compute(self, trajname, \*\*kw)  Compute a hybrid trajectory and store it internally in the 'trajectories' attribute. | source code |

|  |  |
| --- | --- |
| \_validateRegistry(self, obsvars, intvars)  Validate Model's modelInfo attribute. | source code |

|  |  |
| --- | --- |
| \_validateParameters(self) | source code |

|  |  |
| --- | --- |
| \_infostr(self, verbose=1)  Return string information about named sub-model (if given by dsName) at given verbosity level (default 0) | source code |

**Inherited from `Model`**:
`__call__`,
`__copy__`,
`__deepcopy__`,
`__delitem__`,
`__getitem__`,
`__len__`,
`__repr__`,
`__str__`,
`current_defining_args`,
`defaultVars`,
`forceIntVars`,
`forceObsVars`,
`getDSAlgPars`,
`getDSEventActive`,
`getDSEventTerm`,
`getEndPoint`,
`getEndTime`,
`getEventMappings`,
`getTrajEventStruct`,
`getTrajEventTimes`,
`getTrajEvents`,
`getTrajModelName`,
`getTrajTimeInterval`,
`getTrajTimePartitions`,
`has_exact_traj`,
`info`,
`query`,
`renameTraj`,
`resetEventTimes`,
`sample`,
`searchForNames`,
`searchForVars`,
`set`,
`setDSAlgPars`,
`setDSEventActive`,
`setDSEventBisect`,
`setDSEventDelay`,
`setDSEventDir`,
`setDSEventICs`,
`setDSEventInterval`,
`setDSEventPrecise`,
`setDSEventStartTime`,
`setDSEventTerm`,
`setDSEventTol`,
`setICs`,
`setPars`,
`showAuxFnSpec`,
`showAuxSpec`,
`showDSEventInfo`,
`showDef`,
`showEventSpec`,
`showSpec`,
`sub_models`

**Inherited from `Model`** (private):
`_delTraj`,
`_generateParamInfo`,
`_makeDefaultVarNames`,
`_prepareCompute`,
`_set_for_hybrid_DS`,
`_validateVarNames`

**Inherited from `object`**:
`__delattr__`,
`__getattribute__`,
`__hash__`,
`__new__`,
`__reduce__`,
`__reduce_ex__`,
`__setattr__`


|  |  |
| --- | --- |
| Class Variables | [hide private] |

**Inherited from `Model`** (private):
`_needKeys`,
`_optionalKeys`,
`_querykeys`,
`_setkeys`


|  |  |
| --- | --- |
| Properties | [hide private] |

**Inherited from `object`**:
`__class__`


|  |  |
| --- | --- |
| Method Details | [hide private] |

|  |  |
| --- | --- |
| \_\_init\_\_(self, \*a, \*\*kw)  *(Constructor)* | source code |

x.\_\_init\_\_(...) initializes x; see x.\_\_class\_\_.\_\_doc\_\_ for
signature

Overrides: object.\_\_init\_\_: *(inherited documentation)*

|  |  |
| --- | --- |
| cleanupMemory(self) | source code |

Clean up memory usage from past runs of a solver that is interfaced
through a dynamic link library. This will prevent the 'continue'
integration option from being accessible and will delete other data about
the last integration run.

|  |  |
| --- | --- |
| Rhs(self, dsName, t, xdict, pdict=None, asarray=False) | source code |

```
Direct access to a sub-model generator's Rhs function.
Arguments:

  dsName   Name of a sub-model
  t        time (can use 0 for an autonomous system)
  xdict    state dictionary or Point.
  pdict    parameter dictionary or Point
           (optional, default current parameters)
  asarray  (Bool, optional, default False) If true, will return an array
           in state name alphabetical order, else a Point
```

|  |  |
| --- | --- |
| Jacobian(self, dsName, t, xdict, pdict=None, asarray=False) | source code |

```
Direct access to a sub-model generator's Jacobian function (if defined).
Arguments:

  dsName   Name of a sub-model
  t        time (can use 0 for an autonomous system)
  xdict    state dictionary or Point.
  pdict    parameter dictionary or Point
           (optional, default current parameters)
  asarray  (Bool, optional, default False) If true, will return an array
           in state name alphabetical order, else a Point
```

|  |  |
| --- | --- |
| JacobianP(self, dsName, t, xdict, pdict=None, asarray=False) | source code |

```
Direct access to a generator's JacobianP function (if defined).
Arguments:

  dsName   Name of a sub-model
  t        time (can use 0 for an autonomous system)
  xdict    state dictionary or Point.
  pdict    parameter dictionary or Point
           (optional, default current parameters)
  asarray  (Bool, optional, default False) If true, will return an array
           in state name alphabetical order, else a Point
```

|  |  |
| --- | --- |
| MassMatrix(self, dsName, t, xdict, pdict=None, asarray=False) | source code |

```
Direct access to a generator's MassMatrix function (if defined).
Arguments:

  dsName   Name of a sub-model
  t        time (can use 0 for an autonomous system)
  xdict    state dictionary or Point.
  pdict    parameter dictionary or Point
           (optional, default current parameters)
  asarray  (Bool, optional, default False) If true, will return an array
           in state name alphabetical order, else a Point
```

|  |  |
| --- | --- |
| AuxVars(self, dsName, t, xdict, pdict=None, asarray=False) | source code |

```
Direct access to a generator's auxiliary variables
definition (if defined).

Arguments:

  dsName   Name of a sub-model
  t        time (can use 0 for an autonomous system)
  xdict    state dictionary or Point.
  pdict    parameter dictionary or Point
           (optional, default current parameters)
  asarray  (Bool, optional, default False) If true, will return an array
           in state name alphabetical order, else a Point
```

|  |  |
| --- | --- |
| compute(self, trajname, \*\*kw) | source code |

```
Compute a hybrid trajectory and store it internally in the 'trajectories'
attribute.

Arguments (non-keyword):
  trajname   Name of trajectory to create (string)

Arguments (keyword only, all optional):
  force      (Bool, default False) - force overwrite of any trajectory
             stored in this object with the same name
  verboselevel  (int, default 0)
  ics        initial conditions dict or Point
  pars       parameters dict or Point
  tdata      time data (interval as sequence of 2 numeric values)
```

|  |  |
| --- | --- |
| \_validateRegistry(self, obsvars, intvars) | source code |

Validate Model's modelInfo attribute. Now that a HybridModel is
invariably built using ModelConstructor, which already has validation
built in, and also has more complex ways of construction, this method is
defunct.

|  |  |
| --- | --- |
| \_infostr(self, verbose=1) | source code |

Return string information about named sub-model (if given by dsName)
at given verbosity level (default 0)

Overrides: Model.\_infostr: *(inherited documentation)*

  


| Home | Trees | Indices | Help | PyDSTool |
| --- | --- | --- | --- | --- |

|  |  |
| --- | --- |
| Generated by Epydoc 3.0.1 on Fri May 4 15:24:06 2012 | http://epydoc.sourceforge.net |
